# Effects of interobserver and interdisciplinary segmentation variabilities on CT-based radiomics for pancreatic cancer

**DOI:** 10.1038/s41598-021-95152-x

**Published:** 2021-08-11

**Authors:** Jeffrey Wong, Michael Baine, Sarah Wisnoskie, Nathan Bennion, Dechun Zheng, Lei Yu, Vipin Dalal, Michael A. Hollingsworth, Chi Lin, Dandan Zheng

**Affiliations:** 1 Department of Radiation Oncology, University of Nebraska Medical Center, Omaha, NE USA; 2 Department of Radiology, Fujian Medical University Cancer Hospital, Fuzhou, Fujian China; 3 Department of Radiology, University of Nebraska Medical Center, Omaha, NE USA; 4 Department of Biochemistry and Molecular Biology, University of Nebraska Medical Center, Omaha, NE USA; 5 Eppley Institute for Research in Cancer, University of Nebraska Medical Center, Omaha, NE USA

**Keywords:** Cancer imaging, Pancreatic cancer

## Abstract

Radiomics is a method to mine large numbers of quantitative imaging features and develop predictive models. It has shown exciting promise for improved cancer decision support from early detection to personalized precision treatment, and therefore offers a desirable new direction for pancreatic cancer where the mortality remains high despite the current care and intense research. For radiomics, interobserver segmentation variability and its effect on radiomic feature stability is a crucial consideration. While investigations have been reported for high-contrast cancer sites such as lung cancer, no studies to date have investigated it on CT-based radiomics for pancreatic cancer. With three radiation oncology observers and three radiology observers independently contouring on the contrast CT of 21 pancreatic cancer patients, we conducted the first interobserver segmentation variability study on CT-based radiomics for pancreatic cancer. Moreover, our novel investigation assessed whether there exists an interdisciplinary difference between the two disciplines. For each patient, a consensus tumor volume was generated using the simultaneous truth and performance level expectation algorithm, using the dice similarity coefficient (DSC) to assess each observer’s delineation against the consensus volume. Radiation oncology observers showed a higher average DSC of 0.81 ± 0.06 than the radiology observers at 0.69 ± 0.16 (*p* = 0.002). On a panel of 1277 radiomic features, the intraclass correlation coefficients (ICC) was calculated for all observers and those of each discipline. Large variations of ICCs were observed for different radiomic features, but ICCs were generally higher for the radiation oncology group than for the radiology group. Applying a threshold of ICC > 0.75 for considering a feature as stable, 448 features (35%) were found stable for the radiation oncology group and 214 features (16%) were stable from the radiology group. Among them, 205 features were found stable for both groups. Our results provide information for interobserver segmentation variability and its effect on CT-based radiomics for pancreatic cancer. An interesting interdisciplinary variability found in this study also introduces new considerations for the deployment of radiomics models.

## Introduction

Pancreatic cancer is a critical global health care problem. Its low detectability rate and late-stage onset of symptoms contribute to a poor prognosis with a 5-year overall survival rate at 9% for patients diagnosed from 2008 to 2014^1^. Despite decades of research, pancreatic cancer remains an extremely lethal cancer with the highest mortality rate of all major cancers in the US^2^. Radiomics, a new big-data based “omics” branch, has introduced a new direction to facilitate early cancer detection and personalized precision treatment. While it holds potentials to be especially helpful for pancreatic cancer where other research alone yielded limited success, the low contrast and poor conspicuity of pancreatic tumor poses a special challenge. Although there have been developments in abdominal CT imaging, such as dual- and tri-phase contrast imaging and energy spectrum CTs, which have improved the accuracy in defining local tumor extension for pancreatic cancer, tumor segmentation is still particularly challenging^3^.

Radiomics has demonstrated the potential to serve as a tool for the detection, characterization, diagnosis, and prognosis for many cancers^4–7^. With the advancement of machine learning capabilities along with growing interest in personalized medicine, radiomics analysis has become an exciting and current area of research. Radiomics could desirably be applied to pancreatic cancer to aid in early detection and help improve treatment efficacy. The typical radiomic workflow begins with the acquisition of medical images, from which a volume of interest, which is often the tumor, can be segmented for feature extraction. These radiomic features are the input data for statistical or machine learning algorithms to select, integrate, and build predictive models. A critical branch of radiomics research relates to the reproducibility of radiomics analysis^8^. These include challenging issues such as the lack of standardized feature extraction parameters, motion induced effects, volume delineation variation, image acquisition/reconstruction variability, and other factors that lead to non-inherent variability^9–11^. Uncertainties in segmentation are particularly critical since it is one of the upstream steps in the radiomics workflow, therefore affecting all downstream processes. Among these uncertainties relating to segmentation are interobserver variability which has been relatively well-researched for other cancer types such as lung cancer, and interdisciplinary variability, which we wish to introduce in this work.

The effect of interobserver variability on radiomic feature stability and reproducibility has been studied in cancer sites such as lung, breast, glioblastoma, and liver^12–22^. One can expect that the interobserver variability is organ dependent and hence plays a role of varying dominance in the radiomics reproducibility, and therefore needs to be separately characterized for low contrast regions such as the pancreas^23–26^ than high contrast regions such as the lung. In this work, we investigated the segmentation and resulting radiomic feature variations due to the interobserver variability. Contrast enhanced CT images were used for the study as it remains the standard and most used imaging modality for visualizing the pancreas^26^.

In this study, our expert observers included both radiation oncologists and radiologists. These two disciplines also represent the dominating disciplines from which the vast majority of radiomic investigations were conducted^27–31^. Exploring whether an interdisciplinary segmentation variability exists for radiomic research would also be interesting and illuminating. Interdisciplinary variation on segmentation is a new concept and an area of limited study. A recent publication from Nq et al. shows there is significant specialty-dependent variation in contouring post-operative tumor cavities for targeting adjuvant oral cancer therapy^32^. To date, no study has explored the effects of interdisciplinary segmentation variability in the context of radiomics. Thus, in this study we aim to investigate the robustness of radiomic features due to both interobserver and interdisciplinary segmentation variability using contrast-enhanced CT for pancreatic cancer. The study aims both to contribute to the discussion of reproducibility related challenges in the radiomics approach, and to pave ways for radiomics-based applications in pancreatic cancer decision making.

## Results

### Segmentation variability

**Figure 1:**
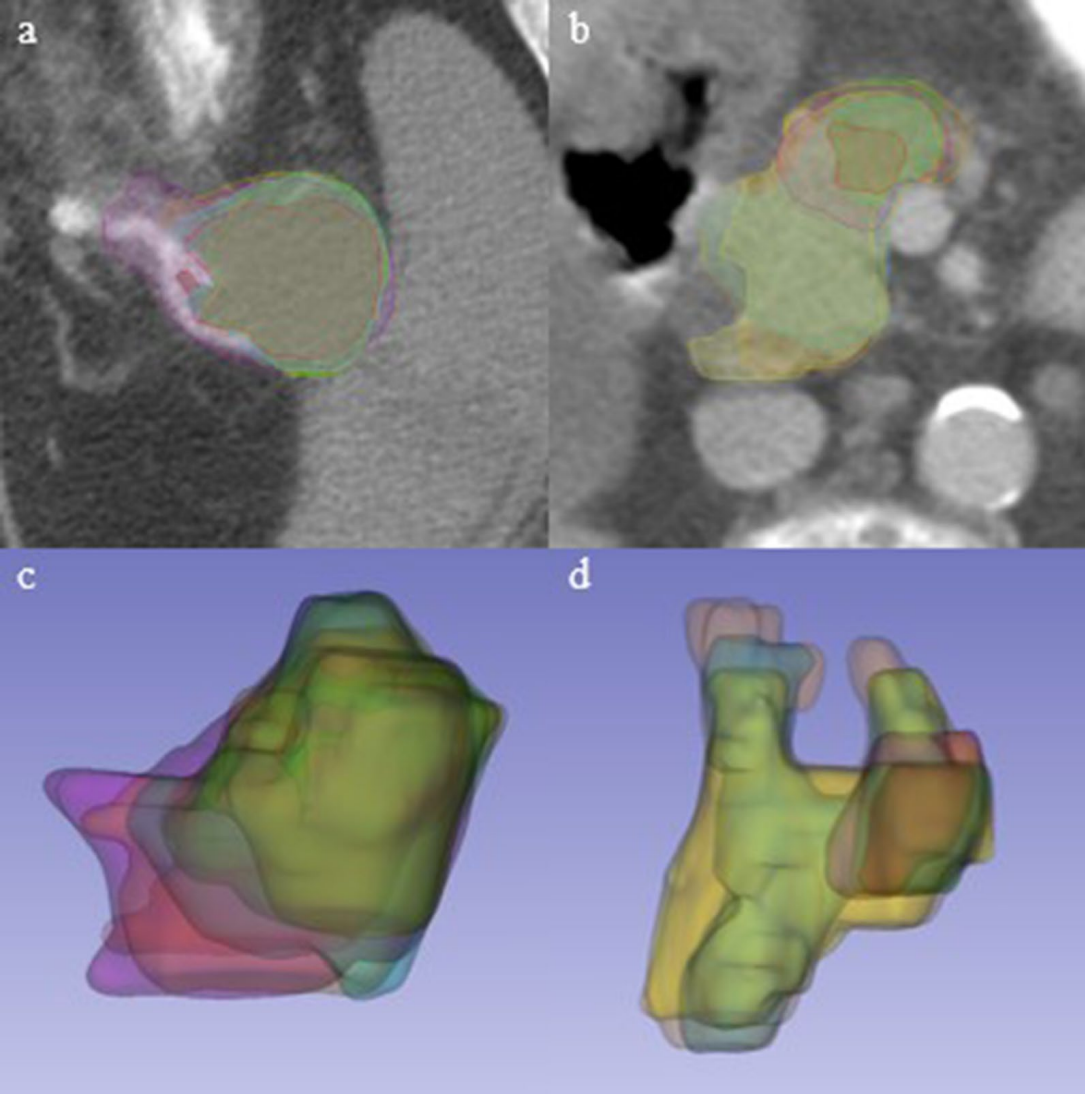
Visualization of contours drawn by all 6 observers. 2D and 3D views of patients with high volume agreement (**a**,**c**) and low agreement (**b**,**d**).

Interobserver and interdisciplinary segmentation variability were observed. Figure 1 illustrates two example cases delineated by all observers with varying degrees of agreement between observers. For the case on the left, a high volume agreement was observed among observers. For the patient on the right, a low agreement was observed. Anecdotally, on this patient, the three radiation oncology observers delineated significantly larger volumes than the three radiology observers. For the patient cohort overall, the radiology observers were also found to contour more conservatively than the radiation oncology observers, yielding a mean volume of. 35.6 ± 15.0 cm^3^ versus 54.1 ± 15.9 cm^3^ (*p* < 0.0001).

**Figure 2:**
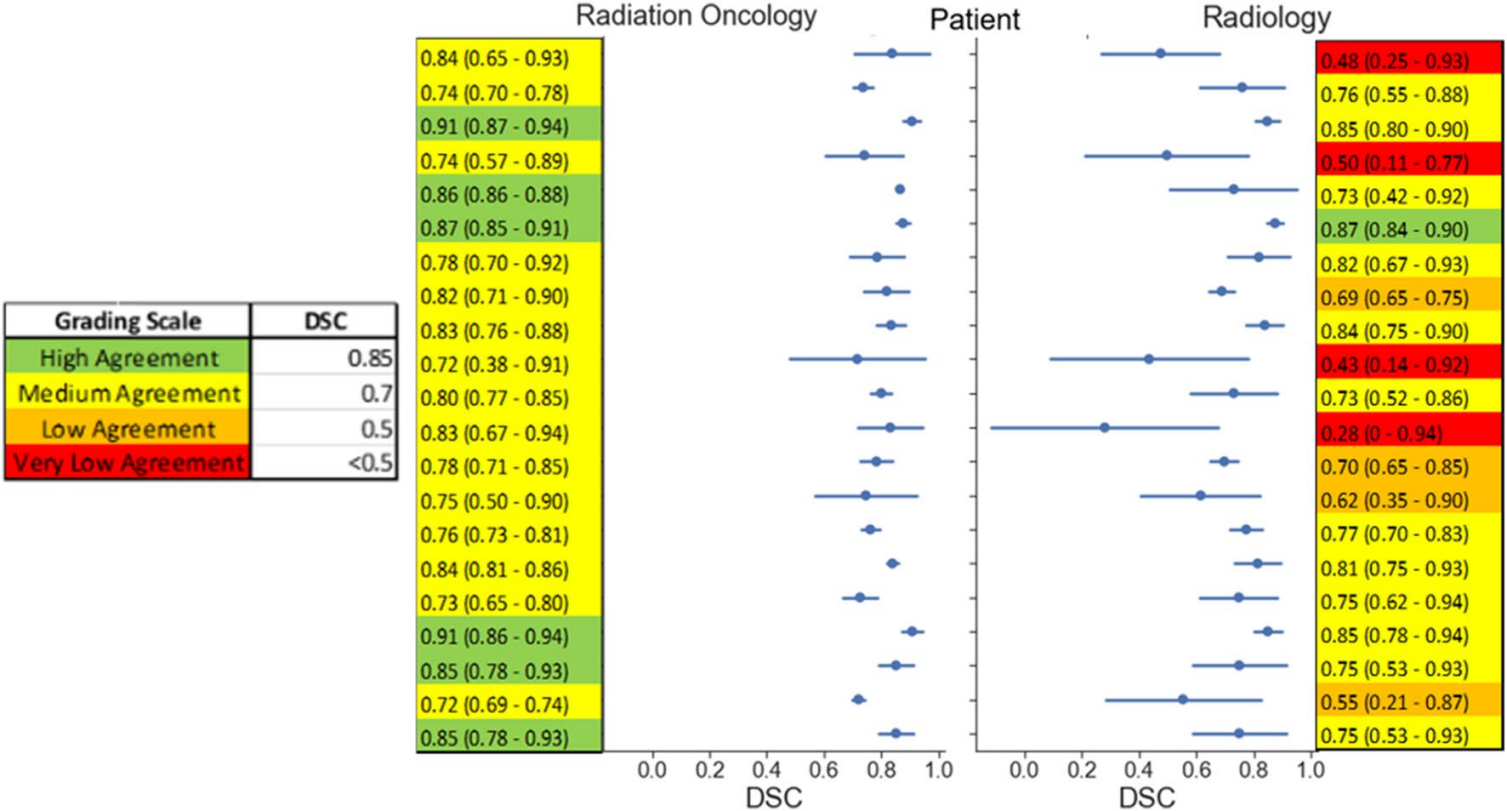
Dice Similarity Coefficient. Center vertical plot shows the mean DSC values (solid dot) with standard deviations (line through dot) for each CT image, separated discipline. Tables adjacent to the plot indicate the numerical values of the mean and standard deviation. DSC values were obtained by pair-wise comparison of the observer contour and STAPLE consensus contour. The legend illustrates the distribution of thresholds used to identify different levels of agreement.

The dice similarity coefficient (DSC) was calculated per patient to quantify the agreement between each observer contour with the consensus contour. Figure 2 shows the distribution of DSC values with mean and standard deviations within each discipline for each patient CT image. The mean DSC for radiation oncology observers and radiology observers were 0.81 ± 0.06 and 0.69 ± 0.16, respectively. Using the grading scale defined in the color legend of Fig. 2, 6 cases of high agreement (DSC > 0.85) were observed for radiation oncology, with no average DSC values falling below medium agreement (DSC < 0.7), compared to the 4 cases of very low agreement from radiology contours (DSC < 0.5). As shown in the distribution plot in Fig. 2, the radiation oncology group showed both a higher average agreement with the consensus segmentation (indicated by higher average DSCs) and a higher agreement with each other (indicated by the tighter standard deviations), when compared with the radiology group. A paired two-tailed independent *T*-test calculated a *p*-value of 0.0038 indicating a statistical significance of the different DSC values between the two disciplines’ observers.

### Radiomic feature robustness

**Figure 3:**
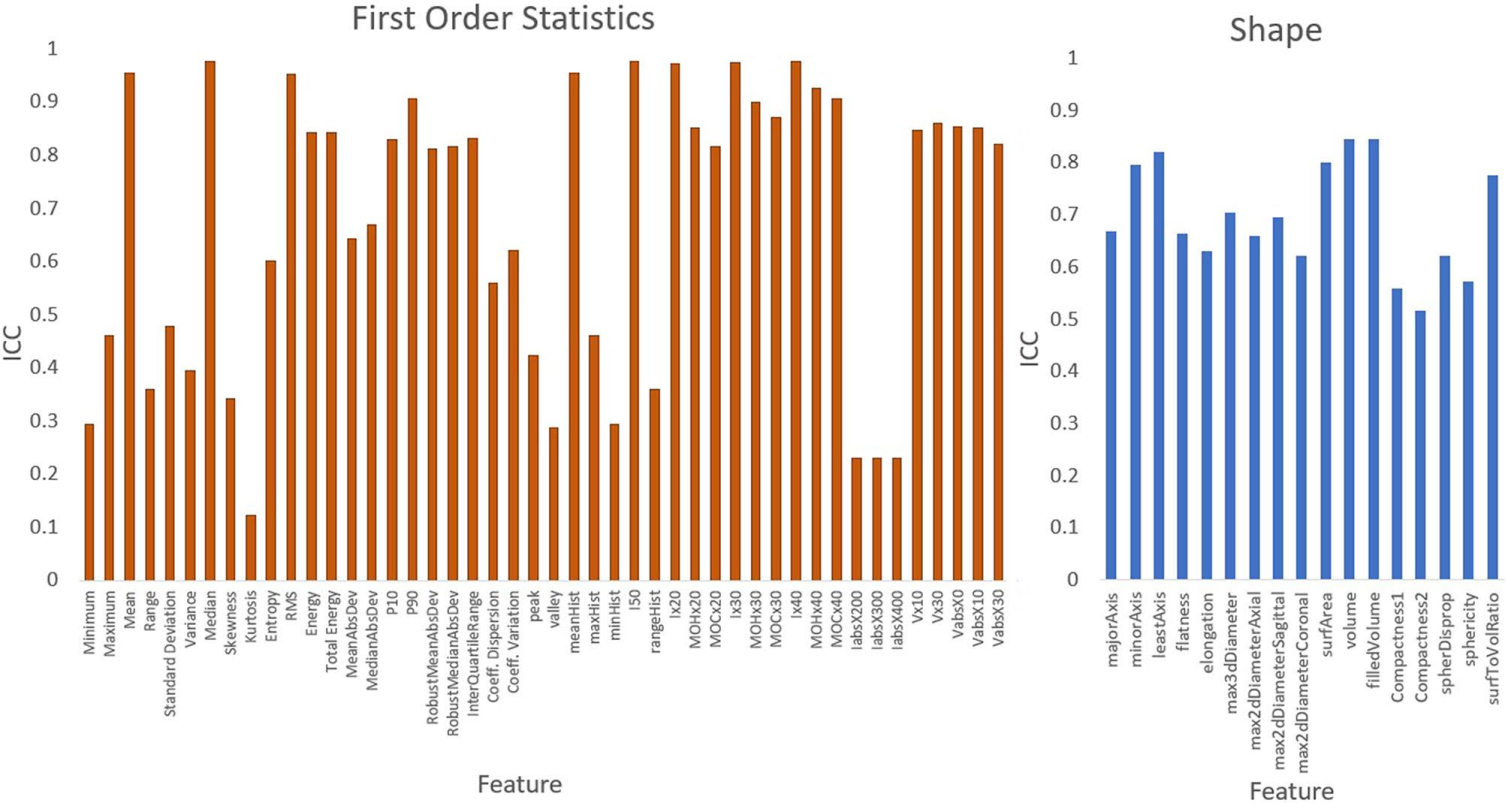
ICC for First order and Shape Features for radiation oncology derived contours only. Features with ICC values > 0.75 were considered robust. Plots of other feature classes are included in Supplement Figures S1–S7 with ICC values from both disciplines.

A total of 1277 features were analyzed for robustness using the intraclass correlation coefficient (ICC) (2,1) calculated both with all 6 observers and separately with the observers of a single discipline. Of these, 143 were extracted from the original image as described in the methods section: 24 first-order, 17 shape, 22 intensity–volume histogram (IVH), and 80 texture features. As examples, the ICC values from radiation oncology contours for first order and shape features are shown in Fig. 3. ICC values from both discipline for all other original features are presented in Supplementary Figures S1–S7.

Comparing the single-discipline observer ICCs, varying degrees of difference were observed for different types of original radiomic features between radiation oncologist observers and radiology observers. For either discipline, relatively low ICCs were observed for some features. Overall, radiomic feature ICCs were lower within the radiology observers than the radiation oncology observers, indicating poorer robustness. Applying ICC ≥ 0.75 as the threshold for features considered robust, 48/143 (34%) original features were robust based on radiation oncology ICCs and 35/143 (24%) were robust based on radiology ICCs.

**Figure 4:**
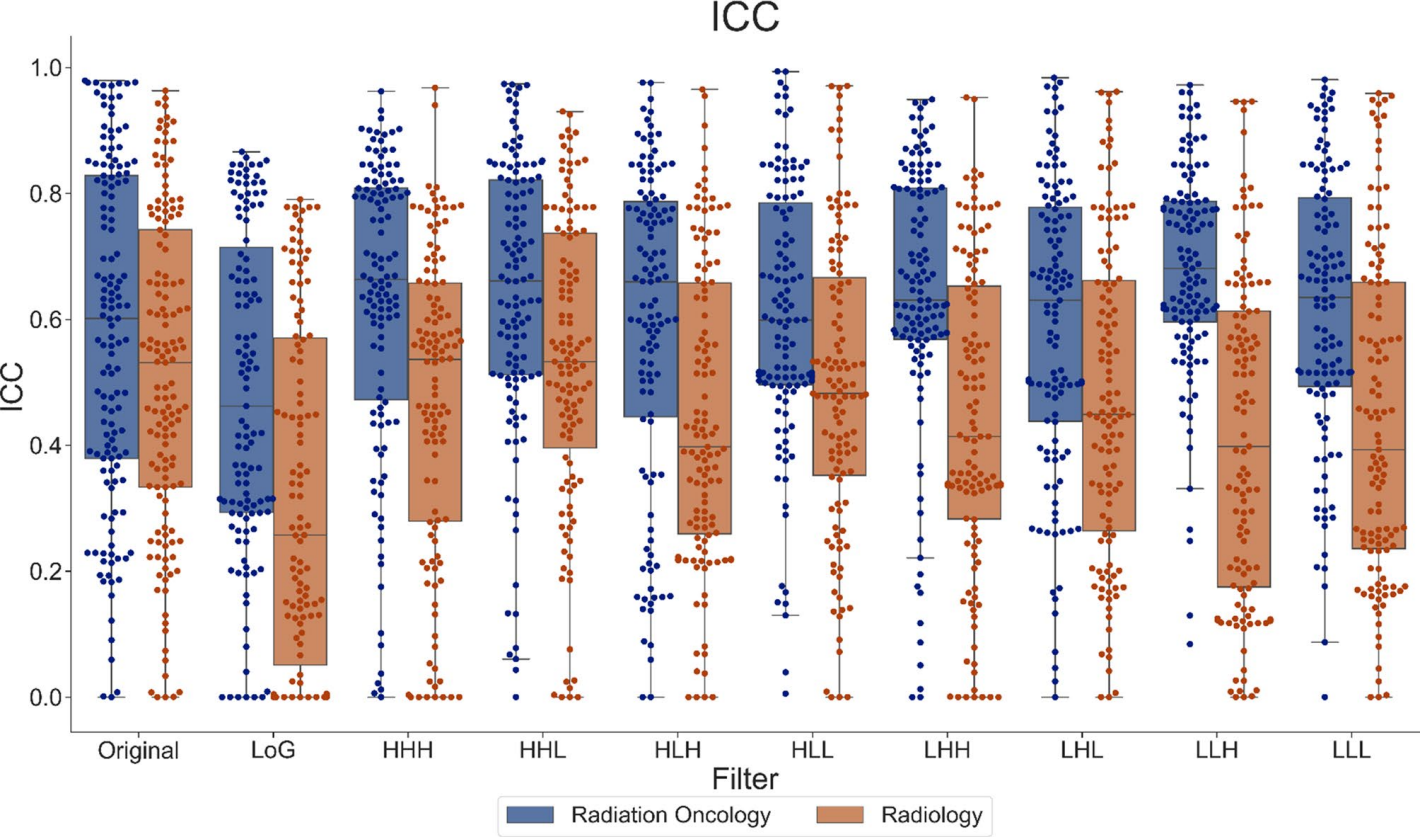
Comparison of ICC of radiomics based on image filter. ICC values for each discipline for radiomic features extracted from each image filter applied illustrates the distribution of ICC values based on image filter type.

To observe the overall distribution of ICC values and compare between the two disciplines for all radiomic features, Fig. 4 shows the spread of single-discipline ICC values for all features based on image filters. Similar to the ICC values for the original features shown in Fig. 4, ICC values derived from either discipline also varied widely for all image types. The higher-order images included Laplacian of Gaussian (LoG), and 8 permutations of 3D wavelets (LLL, HLL, LHL, HHL, LLH, HLH, LHH, and HHH for low or high-pass in each of the three cardinal directions, respectively). The radiation oncology group showed higher average ICCs than the radiology group for all feature categories. Comparing different feature categories, wavelet features showed higher ICCs than original and LoG features, and LoG features showed the lowest ICCs. Out of 1277 features, 448 features (35%) and 214 features (17%) of radiation oncology and radiology derived features were considered robust, respectively. 205 of these features were robust for both disciplines.

The ICC statistics calculated for all features are also listed in Tables 1 and 2 for the radiation oncology and the radiology group, respectively. The feature count and number of robust features are reported for each feature class within each image filter type. As with Figs. 3 and 4, varying robustness were observed for different features and for different feature classes. The top three features with the highest ICCs within each image filter category are listed in Tables 3 and 4 for radiation oncology and radiology groups, respectively. Generally, these top robust features tend to come from certain texture categories such as those based on the run length matrix and the size zone matrix.

**Table 1:** Statistic summary table of radiation oncologist derived stable features.

Filter	Feature class	Total	Robust	% Robust	Filter	Feature class	Total	Robust	% Robust
Original	First order	24	10	41.7	HLL	First order	24	5	20.8
	Shape	17	6	35.3		GLCM	26	6	23.1
	GLCM	26	3	11.5		GLRLM	16	6	37.5
	GLRLM	16	6	37.5		NGTDM/NGLDM	22	6	27.3
	NGTDM/NGLDM	22	4	18.2		GLSZM	16	5	31.3
	GLSZM	16	3	18.8		IVH	22	5	22.7
	IVH	22	16	72.7					
	Total	143	48	33.6		Total	126	33	26.2
LoG	First order	24	9	37.5	LHH	First order	24	11	45.8
	GLCM	26	1	3.8		GLCM	26	8	30.8
	GLRLM	16	6	37.5		GLRLM	16	8	50.0
	NGTDM/NGLDM	22	4	18.2		NGTDM/NGLDM	22	4	18.2
	GLSZM	16	3	18.8		GLSZM	16	4	25.0
	IVH	22	6	27.3		IVH	22	13	59.1
	Total	126	29	23.0		Total	126	48	38.1
HHH	First order	24	16	66.7	LHL	First order	24	6	25.0
	GLCM	26	8	30.8		GLCM	26	8	30.8
	GLRLM	16	8	50.0		GLRLM	16	7	43.8
	NGTDM/NGLDM	22	5	22.7		NGTDM/NGLDM	22	5	22.7
	GLSZM	16	5	31.3		GLSZM	16	4	25.0
	IVH	22	14	63.6		IVH	22	5	22.7
	Total	126	56	44.4		Total	126	35	27.8
HHL	First order	24	9	37.5	LLH	First order	24	12	50.0
	GLCM	26	9	34.6		GLCM	26	12	46.2
	GLRLM	16	8	50.0		GLRLM	16	7	43.8
	NGTDM/NGLDM	22	6	27.3		NGTDM/NGLDM	22	5	22.7
	GLSZM	16	5	31.3		GLSZM	16	6	37.5
	IVH	22	9	40.9		IVH	22	16	72.7
	Total	126	46	36.5		Total	126	58	46.0
HLH	First order	24	14	58.3	LLL	First order	24	11	45.8
	GLCM	26	5	19.2		GLCM	26	5	19.2
	GLRLM	16	8	50.0		GLRLM	16	7	43.8
	NGTDM/NGLDM	22	6	27.3		NGTDM/NGLDM	22	4	18.2
	GLSZM	16	5	31.3		GLSZM	16	3	18.8
	IVH	22	11	50.0		IVH	22	16	72.7
	Total	126	49	38.9		Total	126	46	36.5

Total count of first order, shape, gray-level co-occurrence matrix (GLCM), gray-level run length matrix (GLRLM), neighboring gray tone difference matrix (NGTDM), and neighboring gray-level dependence matrix (NGLDM), gray-level size zone matrix (GLSZM), and IVH features with respective robust features counts are listed. Feature definitions and calculation provided by Computational Environment for Radiological Research (CERR)^35^, as recommended by the imaging biomarker standardization initiative (IBSI)^36^.

**Table 2:** Statistic summary table of radiologist derived stable features.

Filter	Feature class	Total	Robust	% Robust	Filter	Feature class	Total	Robust	% Robust
Original	First order	24	9	37.5	HLL	First order	24	2	8.3
	Shape	17	2	11.8		GLCM	26	3	11.5
	GLCM	26	0	0.0		GLRLM	16	6	37.5
	GLRLM	16	6	37.5		NGTDM/NGLDM	22	4	18.2
	NGTDM/NGLDM	22	3	13.6		GLSZM	16	3	18.8
	GLSZM	16	2	12.5		IVH	22	3	13.6
	IVH	22	13	59.1					
	Total	143	35	24.5		Total	126	21	16.7
LoG	First order	24	2	8.3	LHH	First order	24	2	8.3
	GLCM	26	0	0.0		GLCM	26	0	0.0
	GLRLM	16	2	12.5		GLRLM	16	3	18.8
	NGTDM/NGLDM	22	1	4.5		NGTDM/NGLDM	22	4	18.2
	GLSZM	16	1	6.3		GLSZM	16	3	18.8
	IVH	22	3	13.6		IVH	22	3	13.6
	Total	126	9	7.1		Total	126	15	11.9
HHH	First order	24	6	25.0	LHL	First order	24	5	20.8
	GLCM	26	0	0.0		GLCM	26	3	11.5
	GLRLM	16	4	25.0		GLRLM	16	6	37.5
	NGTDM/NGLDM	22	2	9.1		NGTDM/NGLDM	22	4	18.2
	GLSZM	16	3	18.8		GLSZM	16	3	18.8
	IVH	22	5	22.7		IVH	22	3	13.6
	Total	126	20	15.9		Total	126	24	19.0
HHL	First order	24	8	33.3	LLH	First order	24	2	8.3
	GLCM	26	2	7.7		GLCM	26	0	0.0
	GLRLM	16	6	37.5		GLRLM	16	5	31.3
	NGTDM/NGLDM	22	5	22.7		NGTDM/NGLDM	22	2	9.1
	GLSZM	16	3	18.8		GLSZM	16	2	12.5
	IVH	22	5	22.7		IVH	22	3	13.6
	Total	126	29	23.0		Total	126	14	11.1
HLH	First order	24	5	20.8	LLL	First order	24	5	20.8
	GLCM	26	0	0.0		GLCM	26	0	0.0
	GLRLM	16	3	18.8		GLRLM	16	6	37.5
	NGTDM/NGLDM	22	4	18.2		NGTDM/NGLDM	22	2	9.1
	GLSZM	16	3	18.8		GLSZM	16	2	12.5
	IVH	22	2	9.1		IVH	22	15	68.2
	Total	126	17	13.5		Total	126	30	23.8

Total count of first order, shape, gray-level co-occurrence matrix (GLCM), gray-level run length matrix (GLRLM), neighboring gray tone difference matrix (NGTDM), and neighboring gray-level dependence matrix (NGLDM), gray-level size zone matrix (GLSZM), and IVH features with respective robust features counts are listed. Feature definitions and calculation provided by Computational Environment for Radiological Research (CERR)^35^, as recommended by the imaging biomarker standardization initiative (IBSI)^36^.

**Table 3:** ICC values of top 3 features for radiation oncologist derived contours per filtered image.

Filter	Rank	ICC value	Feature
Original	1	0.979299	rlmFeatS_rlv
	2	0.976673	ivhFeaturesS_Ix40
	3	0.976017	ivhFeaturesS_I50
LoG	1	0.866159	ngldmFeatS_hde
	2	0.856930	ngldmFeatS_gln
	3	0.852354	rlmFeatS_gln
HHH	1	0.962168	szmFeatS_szv
	2	0.931689	szmFeatS_lae
	3	0.919034	rlmFeatS_lre
HHL	1	0.974001	szmFeatS_lae
	2	0.972917	szmFeatS_szv
	3	0.972160	rlmFeatS_lre
HLH	1	0.976052	szmFeatS_szv
	2	0.975441	szmFeatS_lae
	3	0.950179	rlmFeatS_lre
HLL	1	0.993785	szmFeatS_szv
	2	0.993507	szmFeatS_lae
	3	0.976073	rlmFeatS_rlv
LHH	1	0.949382	rlmFeatS_lre
	2	0.944623	rlmFeatS_rlv
	3	0.944030	szmFeatS_szv
LHL	1	0.983789	rlmFeatS_rlv
	2	0.976357	rlmFeatS_lre
	3	0.969907	szmFeatS_szv
LLH	1	0.972301	rlmFeatS_rlv
	2	0.960775	rlmFeatS_lre
	3	0.960107	ngldmFeatS_ldlge
LLL	1	0.980815	firstOrderS_median
	2	0.980274	ivhFeaturesS_I50
	3	0.978090	ivhFeaturesS_Ix30

Feature definitions and calculation provided by Computational Environment for Radiological Research (CERR)^35^, as recommended by the imaging biomarker standardization initiative (IBSI)^36^.

**Table 4:** ICC values of top 3 features for radiologist derived contours per filtered image.

Filter	Rank	ICC value	Feature
Original	1	0.963231	rlmFeatS_rlv
	2	0.951147	szmFeatS_szv
	3	0.943067	szmFeatS_lae
LoG	1	0.848925	ivhFeaturesS_VabsX0
	2	0.790617	ngldmFeatS_hde
	3	0.784417	ivhFeaturesS_Vx30
HHH	1	0.967928	szmFeatS_lae
	2	0.940211	szmFeatS_szv
	3	0.811597	ngldmFeatS_gln
HHL	1	0.930171	szmFeatS_szv
	2	0.925153	ngtdmFeatS_busyness
	3	0.900616	ngldmFeatS_hdlge
HLH	1	0.965352	szmFeatS_szv
	2	0.954827	szmFeatS_lae
	3	0.907794	szmFeatS_lalgle
HLL	1	0.970873	szmFeatS_szv
	2	0.970260	rlmFeatS_rlv
	3	0.967913	szmFeatS_lae
LHH	1	0.952443	szmFeatS_lae
	2	0.949873	szmFeatS_szv
	3	0.836125	rlmFeatS_lre
LHL	1	0.961645	szmFeatS_lae
	2	0.960349	szmFeatS_szvL
	3	0.957630	rlmFeatS_rlv
LLH	1	0.946158	rlmFeatS_rlv
	2	0.945846	szmFeatS_szv
	3	0.945052	szmFeatS_lae
LLL	1	0.959245	ngldmFeatS_gln
	2	0.955016	szmFeatS_szv
	3	0.949218	ivhFeaturesS_I50

Feature definitions and calculation provided by Computational Environment for Radiological Research (CERR)^35^, as recommended by the imaging biomarker standardization initiative (IBSI)^36^.

A Wilcoxon Signed-rank Test was used to measure the statistical difference between radiation oncology and radiology based ICC values for each image filter. All filters indicated a significant difference with *p*-values < 0.0001.

### Feature ranking correlation between two disciplines

**Figure 5:**
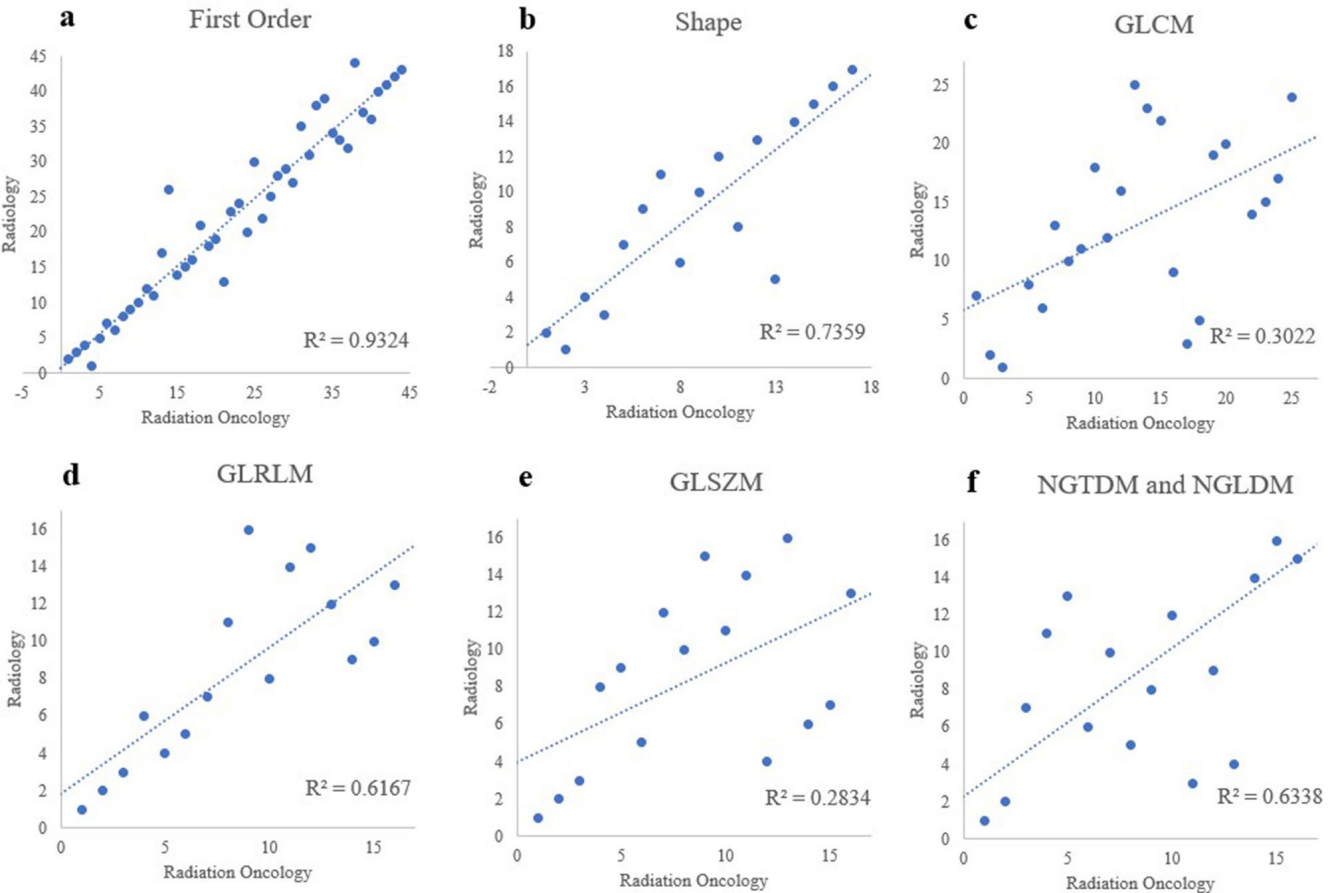
Ranked feature robustness. Features were ranked based on their relative robustness within each feature class for each discipline to indicate potential trends. A slope of 1 would suggest that the relative stability of features tend to be equivalent for both disciplines.

To evaluate relative feature robustness assessed by either discipline, we plotted the original feature ranking within each feature type for the two disciplines in Fig. 5. In other words, in Fig. 5a, for first order features, we plot out their robustness ranking based on radiation oncology observers and radiology observers. The feature rankings are similarly plotted out for other feature classes comparing the two disciplines. A perfect agreement between the two disciplines would result in a 45-degree linear regression of R^2^ = 1. Texture features included gray-level co-occurrence matrix (GLCM), gray-level run length matrix (GLRLM), gray-level size zone matrix (GLSZM), neighboring gray tone difference matrix (NGTDM), and neighboring gray-level dependence matrix (NGLDM). Among different feature classes, the highest degree of robustness ranking agreement was observed for intensity and histogram and shape features, with a R^2^ value of 0.93. Shape features also showed a good agreement with a R^2^ value of 0.74. Texture features showed poorer agreements, with the worst agreements seen for GLCM features and GLSZM features.

## Discussion

To our knowledge, this is the first study addressing the degree of interobserver and interdisciplinary variation in tumor delineation and its effect on CT-derived radiomic feature stability in pancreatic cancer. Using the consensus contours generated based on six expert observers using the Simultaneous Truth and Performance Level Estimation (STAPLE) algorithm, the DSC values between individual observer’s and the consensus contours show considerable interobserver delineation variation for pancreatic cancer. Furthermore, significant interdisciplinary differences are observed between radiation oncology and radiology observer DSCs. These results are also reflected in the large variance of ICC values regardless of image filter and the statistically significant variance between disciplinary-based derived features. Not surprisingly, lower overall ICC values were observed for radiomic features derived from contours with lower DSC values with the consensus volume. This correlation suggests that radiomic feature robustness, and therefore, subsequent radiomic analyses, could be strongly influenced by interobserver delineation variability. But as the radiomic feature showed widely varying degrees of robustness against the interobserver delineation variability, excluding the unstable features in radiomic feature selection appear important for pancreatic cancer model development.

While Belli et al. assessed radiomic feature robustness due to interobserver variation in pancreatic cancer, FDG-PET/CT based contouring was used^20^. Thus, no direct comparison can be drawn with our study as the first of its kind to assess feature robustness due to interobserver variation from contrast-enhanced CT imaging. For low-contrast tumors such as pancreatic cancer, inter-observer delineation uncertainty could be quite different based on anatomy (CT imaging) versus on take-up (PET imaging). Therefore, it is not surprising that our study found lower ICCs than what was previously reported in the PET study. In Luisa Belli et al.’s study evaluating interobserver variability for 73 radiomic features from PET/CT images, 47% of radiomic features were considered robust using an ICC cutoff of 0.80. When considering only features from the non-filtered image in our study, 34% (Radiation Oncology) and 24% (Radiology) were considered robust with an ICC cutoff of 0.75. This difference can be attributed to the utilization of PET imaging, which likely improved tumor conspicuity. Comparing our results for pancreatic cancer with previous studies investigating radiomic feature robustness against segmentation variability in other sites such as breast, non-small cell lung cancer, glioblastoma, and liver, the ICCs are also considerably lower^12–22^. In comparison, Pavic et al. found 90% and 56% of radiomic features considered stable with an ICC cutoff of 0.80 despite interobserver variation in non-small cell lung cancer and head and neck squamous cell cancer, respectively^12^. The results indicate that although all these studies confirm that contour variability impacts radiomic feature stability and features show varying degrees of robustness, interobserver variability will likely be more dominating among different sources of uncertainty to impact radiomic feature reproducibility for pancreatic cancer than for other high-contrast cancers such as lung cancer.

Interestingly, our investigation also revealed a significant interdisciplinary delineation variation. This is to our knowledge the first study to investigate the existence of a specialty difference in segmentation for radiomics. Our results agree with previous report from Nq et al. on radiotherapy target delineation for post-operative head and neck cancer patients^32^ that a specialty difference does exist. We observe that the tumor volumes from the radiation oncology group tend to be larger than those from the radiology group. Despite our efforts to standardize the contouring experience, this could have resulted from radiation oncologists’ tendency to “not miss the target” in their training despite the common contouring guideline in this study to exclude any uncertain area. In addition, we found higher consistency among the radiation oncology group than among the radiology group, possibly stemming from the practice difference that volume segmentation is a routine activity in radiation oncology, but in radiology volume labeling is more important than whole volume segmentation. Among different classes of original radiomic features, the relative agreement between the two disciplines is better for first-order features and shape features than the texture features. These novel findings are important as vast majority of the current radiomic models are developed based on delineated volumes either from radiology or radiation oncology. Such interdisciplinary delineation uncertainties should be considered when the segmentation is done by a different specialty in model deployment than in model development. Some studies have suggested the use of semiautomatic segmentation to improve radiomic feature robustness against volume delineation^14–16^. However, the current semiautomatic segmentation methods’ effectiveness in low contrast regions such as that of pancreatic cancer in CT imaging may be more challenging.

There are some limitations to our study to consider. Intraobserver variability was not investigated in our study. The threshold ICC value of 0.75 was selected based on literature investigating radiomic feature robustness due to interobserver variation in other tumor sites^12–21^. With ICC thresholds between 0.7 and 0.9 seen in the literature, our study chose a threshold of 0.75 on the lower end, adding a small conservative margin to account for the increased difficulty in contouring pancreatic cancer compared to those in the aforementioned studies. The significance of this threshold with respect to its effect on radiomics analysis, however, has not been investigated. Additionally, our study evaluated feature robustness, but its effect on the latter phases of radiomics analysis and how best to address it were not included. Also, this investigation is based solely on CT images while MRI and PET images also provide additional information and are sometimes fused to CT for pancreatic tumor evaluation. However, as CT remains the dominating radiological imaging modality in clinical practice for pancreatic cancer, we chose to focus the current study on CT-based radiomics. In our study, CT scans of limited scanner types and acquisition parameters were selected to isolate the observer variability from variabilities of imaging factors. Similarly, a fixed window/level setting was imposed in our study, ensuring different observers’ contours were not affected by varying window/level settings individual observers may choose. Yet by doing so, our findings reflect the results obtained with our specific image acquisition, reconstruction, and display parameters, and may need validation before generalized to other cases. In addition, our radiomic feature results from 5 mm isotropic voxels could be affected by edge and partial volume effects, though the tumor volumes are relatively large with a mean volume of 50.4 cm^3^.

Overall, the results of our investigation contribute to the conversation necessitating more rigorous evaluation of volume reproducibility prior to radiomic feature analysis. Our site-specific findings for pancreatic cancer are important as feature robustness against segmentation uncertainty will likely play a more dominating role in the reproducibility of radiomics for such cancers. The novel discovery on interdisciplinary variations also introduces new considerations for the deployment of radiomics-based predictive models.

## Methods

### Study cohort

Under the approval of the Institutional Review Board of University of Nebraska Medical Center (IRB#091-01-EP and IRB#127-18-EP), the diagnostic contrast-enhanced CT scans from 21 pancreatic cancer patients were used for this study. The patients whose images were investigated in this study were all enrolled with informed consents in the Rapid Autopsy Pancreas Program at University of Nebraska Medical Center. To date, the program has collected unique tissue specimens within hours of death from over a hundred enrolled pancreatic cancer patients over the past decade. Retrospective analyses in this study were performed in accordance with the relevant guidelines and regulations as approved by the Research Ethics Committee of University of Nebraska Medical Center. From the patients with available contrast-enhanced CT images at the time of diagnosis, we selected the maximum number of patients imaged with the same line of CT scanners from a single vendor and acquired with the same acquisition protocol and slice thickness to minimize the effect of other uncertainty-contributing factors. This resulted in the 21 patients used in the study. These 21 patients included 1 stage IIA, 2 stage IIB, 1 stage III, and 17 stage IV patients.

### Image acquisition and volume segmentation

For the 21 patients included in the study, the image acquisition was performed using one of the following three CT scanner models: Lightspeed VCT, Lightspeed Pro 16, and Lightspeed RT16 (GE Healthcare, Boston, Massachusetts, USA). Patients received ISOVUE injection with bolus triggering arterial phase imaging about 30 s and venous phase about 60 s after injection. A slice thickness of 5 mm was used for all patient acquisitions, and the in-plane resolution was between 0.6 and 0.9 mm.

Six expert observers, comprised of three radiation oncologists and three radiologists, each contoured the entire cohort. The three radiation oncologists have 21, 8, and 6 years of experience within their specialty, respectively, and the three radiologists have 19, 7, and 4 years of experience within their specialty, respectively. All observers in the study are experts in assessing/treating pancreatic tumors as their clinical and/or research specialization. For each patient, the tumor was contoured using the iPlan software (Brainlab AG, Feldkirchen, Germany). To standardize the delineation for individual observers, the following segmentation instructions were given:

- Tumor: Only contour what you feel certain is the gross tumor. Where it is uncertain, exclude.
- Exclude major vessels, stents, markers, and lymph nodes, if applicable.
- A window width of 400 with level at 50 has been preset for all cases. Do not change the window/level.
- Use only the assigned CT for contouring. Do not rely on the help of MR, or other CT data.
- Complete contours independently.

All contours were visually inspected by a non-observer investigator. In a couple of instances, an individual contour deviated substantially from that of other observers’. The observer was asked to review their contour, with the option to edit if so chose, while still blinded from other observers’ contours.

### Consensus volume generation and segmentation variability assessment

For each patient, a consensus volume was created using the STAPLE algorithm as a ground truth surrogate^33^. The STAPLE algorithm creates a consensus volume based on the volume delineations from all 6 observers.

The dice similarity coefficient (DSC) was used to quantify the degree of volume overlap between two volumes^34^. The calculation of the DSC value is defined in Eq. 1,

1 $$DSC = \frac{2\left| A \right| \cap \left| B \right|}{{\left| {A\left| + \right|B} \right|}}$$

where of A and B are the two volumes for which the DSC is to be calculated, and $\cap$ indicates the intersection of the two volumes. The DSC is between 0 and 1, with 1 indicating two identical contours and 0 indicating two completely different contours.

Because the DSC requires a pair-wise comparison, it was calculated for each observer contour to quantify the overlap with the consensus volume. A value of 1 indicates complete spatial overlap with the consensus volume, whereas a DSC of 0 indicates no overlap. For illustration purposes, the following scale was used to categorize the level of volume agreement: DSC ≥ 0.85 [High Agreement], 0.85 > DSC ≥ 0.70 [Medium Agreement], 0.7 > DSC ≥ 0.5 [Low Agreement], DSC < 0.5 [Very Low Agreement]. All DSC values were calculated using the Computational Environment for Radiological Research (CERR) in Matlab R2018b^35^.

### Image processing and radiomic feature extraction

For this study, a panel of 1277 radiomic features were calculated using an adapted version of CERR implemented in Matlab R2018b. These features consisted of: first order (n = 24), shape (n = 17), texture (n = 80), and intensity–volume histogram (IVH) (n = 22), on the original image and with 9 filters applied: Laplacian of Gaussian (LoG, n = 126), and 8 permutations of 3D wavelets (LLL, HLL, LHL, HHL, LLH, HLH, LHH, and HHH totaling n = 1008). As the shape features stay invariant with image filters, they were excluded in counting LoG and wavelet features. Individual calculated values can be found in the Supplementary Dataset for all features extracted using CERR, which followed the recommendations provided by the image biomarker standardization initiative (IBSI)^36^. Before feature extraction, the voxels were made isotropic by resampling the images to 5 × 5 × 5 mm^3^ voxels using sinc interpolation. Images were discretized using a bin width of 25 and the texture matrices were calculated for all three dimensions (resulting in 26 directions, or 13 symmetrical directions) with a voxel offset of 1 for neighboring voxels.

### Radiomic feature robustness evaluation

The intraclass correlation coefficient (ICC) was used to quantitatively evaluate the robustness of radiomic features due to interobserver variations. An ICC (2,1) was selected to account for two-way random effects with single measurements when assessing the absolute agreement^37–39^. ICC values were also calculated separately based on discipline (radiation oncology vs. radiology) to assess interdisciplinary effects.

2 $$ICC (2,1) = \frac{{{\text{MS}}_{{\text{R}}} - {\text{MS}}_{{\text{E}}} }}{{{\text{MS}}_{{\text{R}}} + ({\text{k}} - 1){\text{MS}}_{{\text{E}}} + \frac{{\text{k}}}{{\text{n}}}\left( {{\text{MS}}_{{\text{C}}} - {\text{MS}}_{{\text{E}}} } \right)}}{ }$$

Radiomic features with ICC values greater than 0.75 were considered reproducible and robust. All ICC values were calculated using the ‘irr’ package in RStudio^40^.

### Ethics approval

The study was approved by the Institutional Review Board of University of Nebraska Medical Center (IRB#091-01-EP and IRB#127-18-EP).

## Conclusion

Volume segmentation variability affects radiomic feature stability for CT-based radiomics studies in pancreatic cancer, as has been shown for other cancer sites. Considerably lower interobserver ICCs were found than for high-contrast cancer sites, suggesting a more dominating role segmentation uncertainty plays in radiomics for pancreatic cancer. A novel interdisciplinary variability is also observed on segmentation, introducing new considerations for the deployment of radiomics-based predictive models.

## Supplementary Information

Supplementary Figures.

Supplementary Information.
