# Supplementary material for: Effects of interobserver and interdisciplinary segmentation variabilities on CT-based radiomics for pancreatic cancer
Source: Sci Rep. 2021 Aug 11;11:16328. doi: 10.1038/s41598-021-95152-x (PMC8357939; doi:10.1038/s41598-021-95152-x)
Supplement: Supplementary file 1 — Supplementary Figures. [file 41598_2021_95152_MOESM1_ESM.pdf]

# Effects of interobserver and interdisciplinary segmentation variabilities on CT-based radiomics for pancreatic cancer

## Supplementary Materials

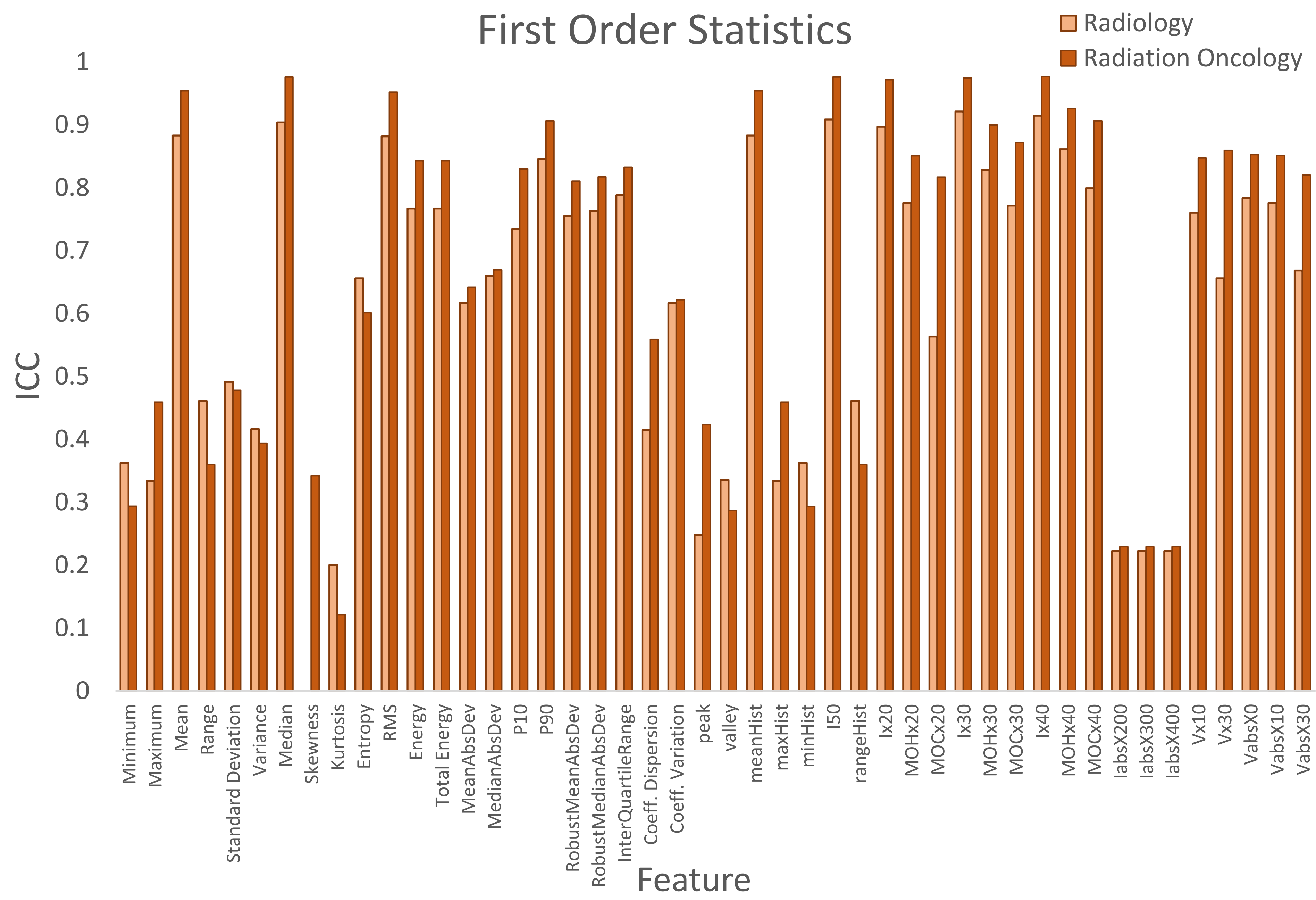


**Figure S1.** Histogram distribution of intraclass correlation coefficient (ICC (2,1)) for first-order statistics features from radiation oncology and radiology derived contours.

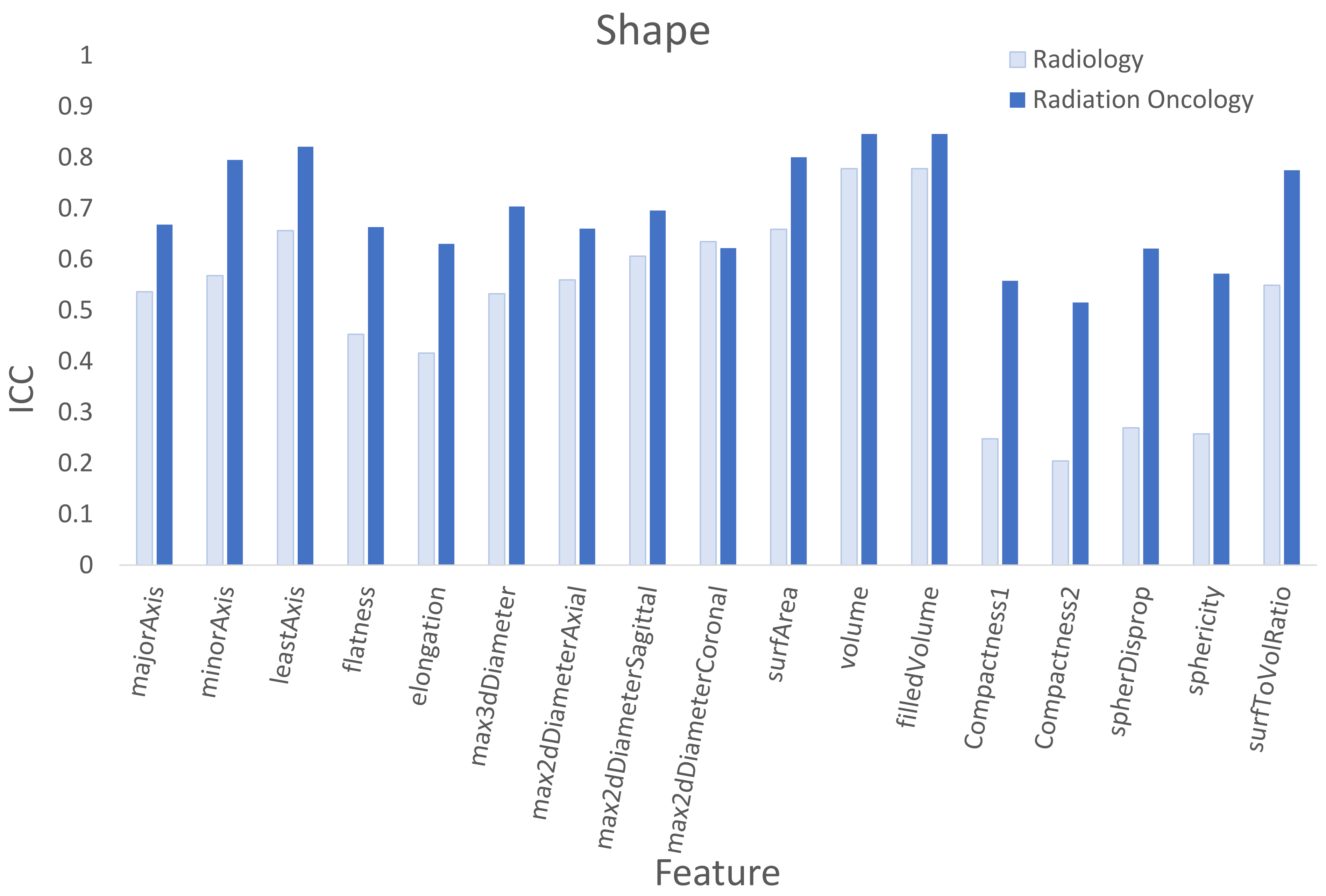


**Figure S2.** Histogram distribution of intraclass correlation coefficient (ICC (2,1)) for shape features from radiation oncology and radiology derived contours.

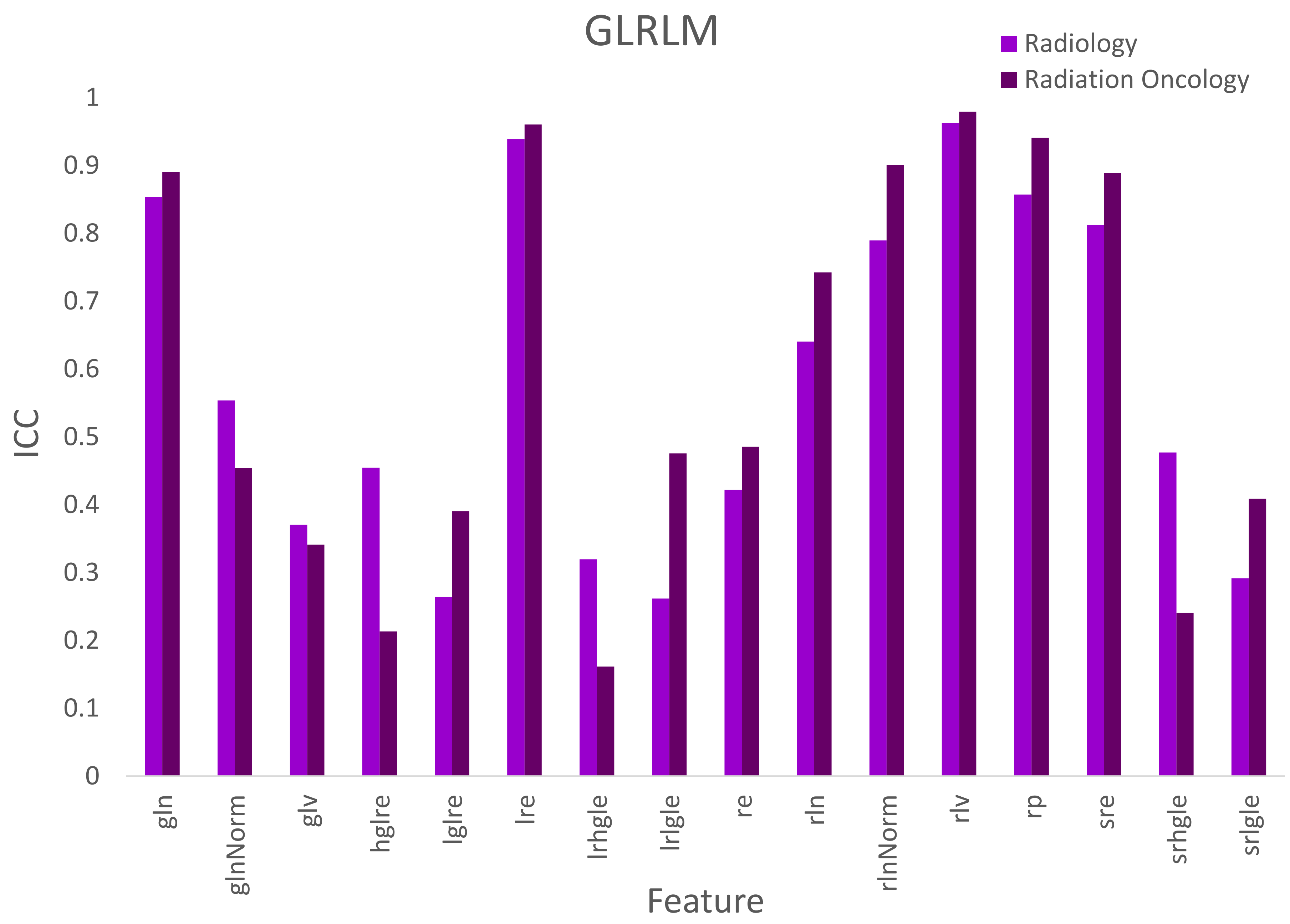


**Figure S3.** Histogram distribution of intraclass correlation coefficient (ICC (2,1)) for gray level run length matrix (GLRLM) features from radiation oncology and radiology derived contours.

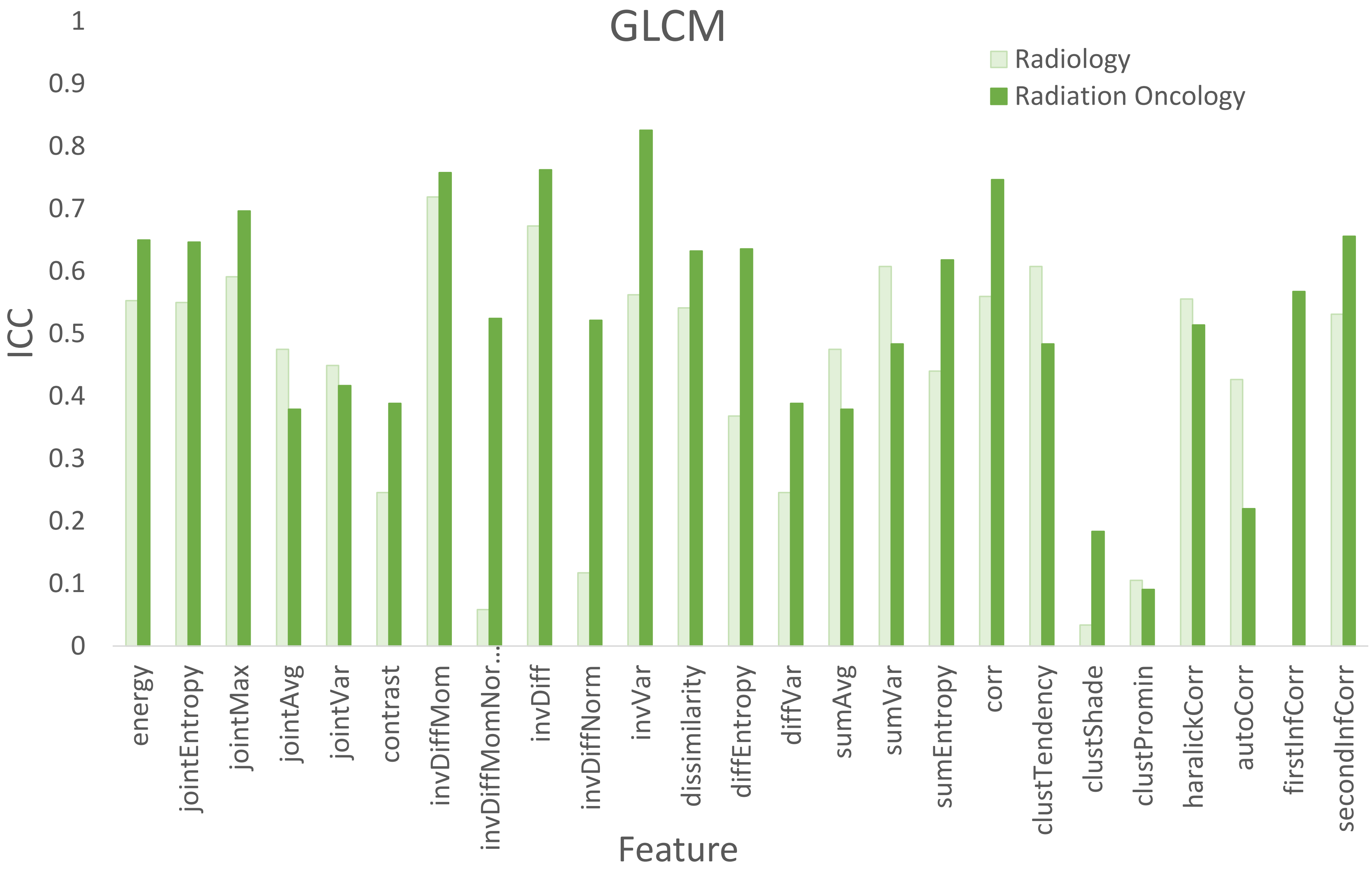


**Figure S4.** Histogram distribution of intraclass correlation coefficient (ICC (2,1)) for gray level co-occurrence matrix (GLCM) features from radiation oncology and radiology derived contours.

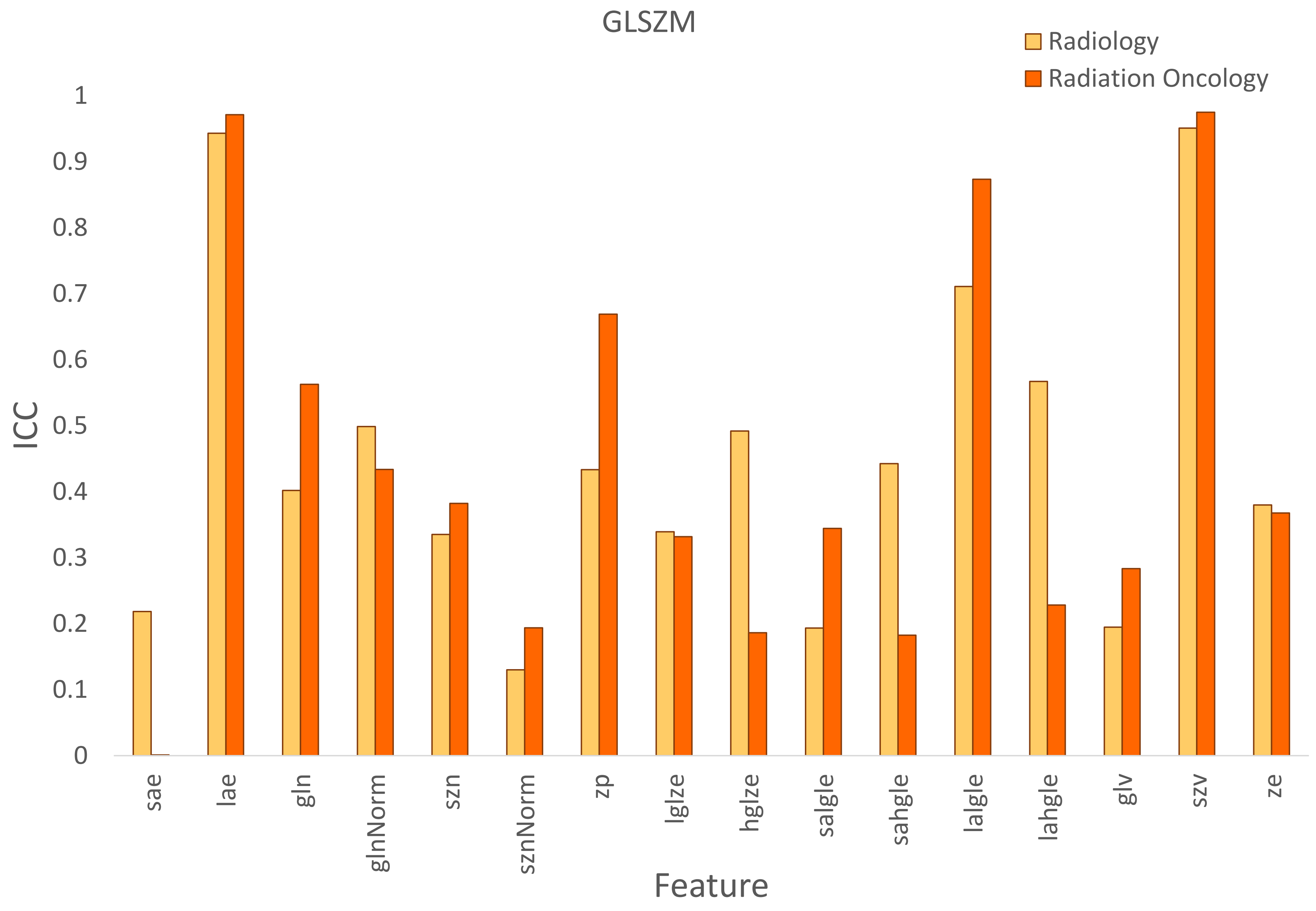


**Figure S5.** Histogram distribution of intraclass correlation coefficient (ICC (2,1)) for gray level size zone matrix (GLSZM) features from radiation oncology and radiology derived contours.

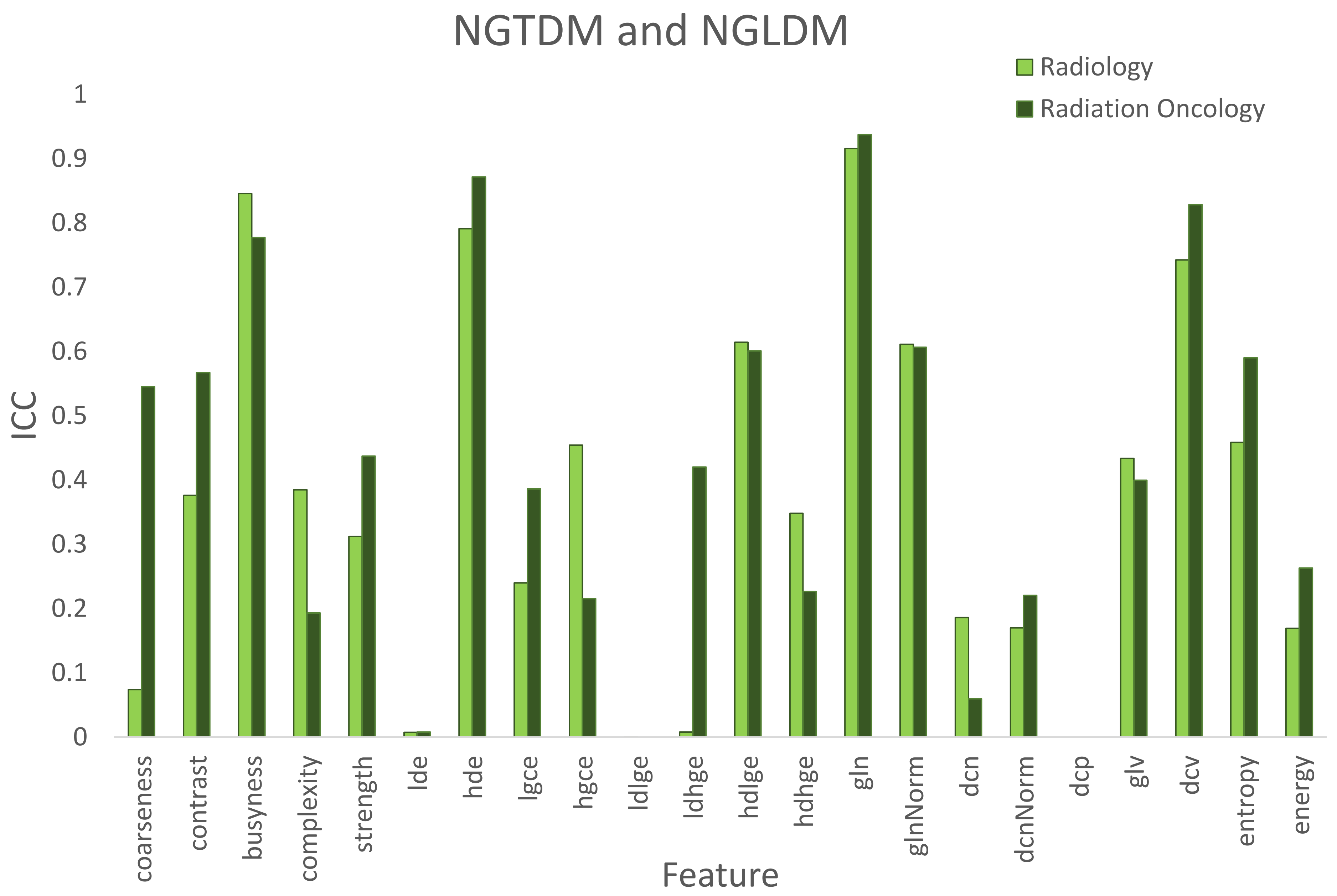


**Figure S6.** Histogram distribution of intraclass correlation coefficient (ICC (2,1)) for neighboring gray tone difference matrix (NGTDM) and neighboring gray level dependence matrix (NGLDM) features from radiation oncology and radiology derived contours.

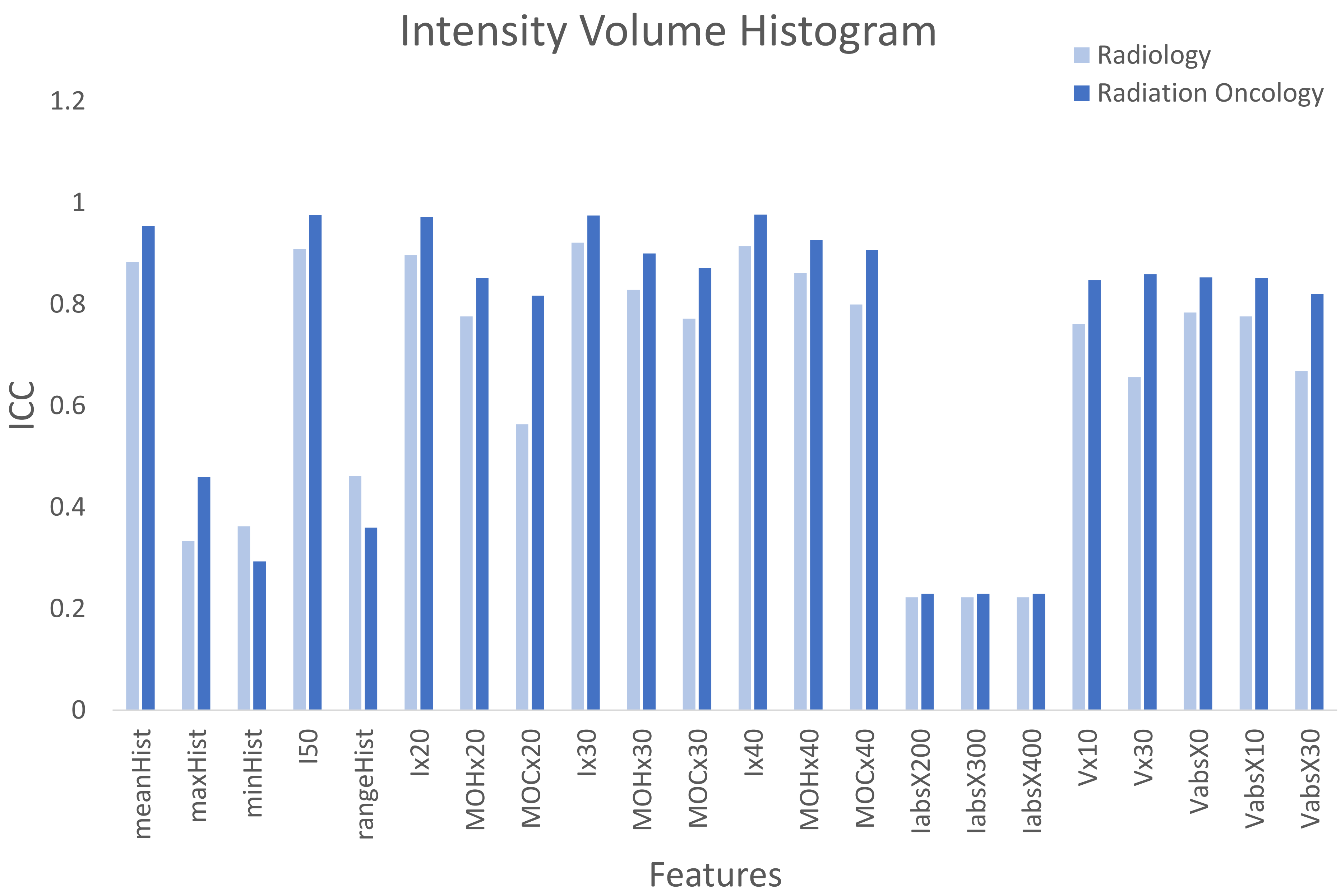


**Figure S7.** Histogram distribution of intraclass correlation coefficient (ICC (2,1)) for intensity volume histogram features from radiation oncology and radiology derived contours.
